# ParsEval: parallel comparison and analysis of gene structure annotations

**DOI:** 10.1186/1471-2105-13-187

**Published:** 2012-08-01

**Authors:** Daniel S Standage, Volker P Brendel

**Affiliations:** 1 Department of Genetics, Development, and Cell Biology, Iowa State University, Ames, Iowa 50011, USA; 2 Department of Biology, Indiana University, Bloomington, Indiana 47405,, USA; 3 School of Informatics and Computing, Indiana University, Bloomington, Indiana 47405, USA

## Abstract

**Background:**

Accurate gene structure annotation is a fundamental but somewhat elusive goal of genome projects, as witnessed by the fact that (model) genomes typically undergo several cycles of re-annotation. In many cases, it is not only different versions of annotations that need to be compared but also different sources of annotation of the same genome, derived from distinct gene prediction workflows. Such comparisons are of interest to annotation providers, prediction software developers, and end-users, who all need to assess what is common and what is different among distinct annotation sources. We developed ParsEval, a software application for pairwise comparison of sets of gene structure annotations. ParsEval calculates several statistics that highlight the similarities and differences between the two sets of annotations provided. These statistics are presented in an aggregate summary report, with additional details provided as individual reports specific to non-overlapping, gene-model-centric genomic loci. Genome browser styled graphics embedded in these reports help visualize the genomic context of the annotations. Output from ParsEval is both easily read and parsed, enabling systematic identification of problematic gene models for subsequent focused analysis.

**Results:**

ParsEval is capable of analyzing annotations for large eukaryotic genomes on typical desktop or laptop hardware. In comparison to existing methods, ParsEval exhibits a considerable performance improvement, both in terms of runtime and memory consumption. Reports from ParsEval can provide relevant biological insights into the gene structure annotations being compared.

**Conclusions:**

Implemented in C, ParsEval provides the quickest and most feature-rich solution for genome annotation comparison to date. The source code is freely available (under an ISC license) at http://parseval.sourceforge.net/.

## Background

It was only a decade ago when annotating a eukaryotic genome required years of extensive collaboration and millions of dollars of investment. Since then, the tremendous rate at which the cost of DNA sequencing has been dropping as well as increased accessibility to gene prediction software are placing genome sequencing and annotation well within the reach of most single investigator biology laboratories. As a result, proliferation of distinct annotation sets corresponding to the same genomic sequences is becoming increasingly common. Annotation sets for a particular genome can accumulate in a variety of scenarios. When developing gene prediction software, it is common to test the software on a genomic region for which a high-quality reference is available, running and re-running the software and comparing the resulting predictions against the reference. Community groups providing annotation for species- or clade-specific genomes typically release updated annotations following the initial release. Affordable transcriptome sequencing provides individual labs with data to specifically improve annotations for particular genes of interest, for example with respect to alternative splicing. In each of these scenarios, multiple annotations associated with a common set of genomic sequences require comparative assessment.

A variety of comparison methods exist, but none can fully address the growing needs of the community (see Table 1). Manual comparison approaches can trivially be ruled out as slow, tedious, error prone, and hopelessly unscalable. Although genome browsers have had a huge impact by making gene annotations accessible to a wide variety of scientists, they likewise do little to provide the automation and precision needed in whole-genome annotation comparisons. Large genome sequencing projects and centers have certainly developed in-house scripts and pipelines over the years to address this need. However, these pipelines are typically not standardized, not openly shared, and do not migrate well.

**Table 1:** Annotation comparison methods

**Method**	**Pros**	**Cons**			
Manual comparison	minimal overhead	extremely tedious; error prone; unscalable			
Genome browser	intuitive interface; visual assessment of individual loci	visual assessments imprecise; extensive overhead; little or no automation			
Eval	detailed statistics; visual assessment of statistic distributions; scales fairly well for large data sets; can compare multiple predictions to a single reference	older software; relatively slow; only summary statistics are reported, while stats for individual loci are discarded			
ParsEval	detailed statistics provided, not only as a summary but for individual loci as well; scales well for large data sets; fast, efficient, and portable	only capable of comparing a single pair of annotations			

Various approaches to comparing alternative sources of gene structure annotations, with a brief description of the associated pros and cons.

Tools such as the Eval package [1] and the GFPE program [2] represent some of the earliest efforts to provide a reusable, easy-to-use annotation comparison tool to the community. Eval in particular stands out based on the amount of detail provided by its reported comparison statistics and by the ability to visualize the distributions of these statistics. Eval takes as input annotation files in Gene Transfer Format (GTF) and calculates a rich set of descriptive statistics summarizing the differences between the annotations. Because whole-genome annotations typically include thousands (or tens of thousands) of genes, these statistics are intended to condense the information into a comprehensive yet concise summary (at the resolution of entire sequences or sets of sequences), facilitating targeted improvement of gene prediction software. Unfortunately, this condensing process discards large amounts of valuable information at the resolution of individual gene loci, making the tool unsuitable for analyses that target a particular gene, sets of genes, or gene loci with characteristics of interest from within a larger set of genes. Such locus-resolution comparisons are useful not only to software developers and annotation producers who need to know whether their software has distinct advantages or disadvantages, e.g., favoring long over shorter gene models on average, or failing in untranslated region (UTR) prediction, but they are of primary interest for specialists concerned with a particular gene family or pathway.

Motivated by a need for genome-scale evaluations with locus-scale detail, we developed ParsEval, a program for comparing and analyzing distinct sets of gene structure annotations for the same input sequences. The program is designed to incorporate all of the benefits of existing methods while addressing their shortcomings. ParsEval identifies differences in exon/intron assignments and in coding sequence (CDS) and UTR designations, at both feature-level (exon, CDS segment, UTR segment) and nucleotide-level resolution. The output consists of a set of commonly used statistics that provide quantitative measures of agreement when comparing predicted gene structures against a standard reference [3-5]. This output is presented in a detailed report for each gene locus, supplemented with genome browser styled graphics to enable additional visual assessment and analysis of the annotations. The statistics are also presented in a single summary report that aggregates the statistics across all loci, providing a condensed high-level view of the similarity between the two sets of annotations. For gene loci that include alternatively spliced genes or overlapping genes (or both), ParsEval determines the optimal matching of reference transcripts to prediction transcripts, and additionally reports any novel transcript predictions that have been identified.

## Implementation

### Overview

ParsEval is a command-line tool for gene annotation comparison and analysis. The program takes as input a pair of gene structure annotations corresponding to the same sequence (in GFF3 format [6]), analogous to two separate annotation tracks one might see in a genome browser. For comparison purposes, the first set of annotations is treated as the *reference* while the other is treated as the *prediction*, although ParsEval makes no assumptions regarding the respective quality of the two annotation sets. The output of the program is a set of reports containing common comparison statistics intended to highlight relevant similarities and differences between the two sources of annotation.

ParsEval first loads the annotation data into memory, identifies start and end coordinates for gene loci, and associates each gene annotation with a single locus. Next, the program does a comparative assessment of the gene annotations for each locus, calculating and storing a variety of informative similarity statistics. Finally, ParsEval generates reports providing a detailed readout of these statistics.

Implemented in ANSI C, ParsEval is fast, memory efficient, and portable, designed to run on all POSIX-compliant UNIX systems (Linux, Mac OS X, Cygwin, Solaris, etc.). Most of the analysis code is implemented with shared memory parallelization, providing additional performance gains when running on multicore processors that are becoming increasingly common in commodity hardware. ParsEval’s only external dependency is the GenomeTools library [7], which provides an API for generating annotation graphics with AnnotationSketch [8], as well as implementations of a variety of data parsers and dynamic data structures.

### Gene locus identification

Comparative analysis of two sets of gene annotations requires determining how annotations from one set correspond to annotations from the other, as well as the genomic coordinates (the *gene locus*) that should be considered in each comparison. For rare cases in which a single reference annotation and a single prediction annotation line up perfectly, determining the gene locus and the corresponding genes is trivial. However, in most cases this task is complicated a variety of factors. For example, a single gene prediction workflow may annotate multiple genes at a single location, so one must determine how to associate these annotations with corresponding annotations from an alternative source. Furthermore, when one or more gene annotations from one source overlap with multiple annotations from another source, one must determine how to compare these gene annotations and which coordinates to include in the comparison.

**Figure 1:**
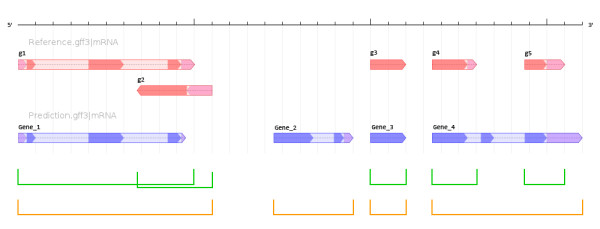
**Associating Annotations with Gene Loci.** The black bar provides a scale corresponding to a genomic region for which two sets of annotations are available. Reference annotations for gene structure are represented with red glyphs, while prediction annotations are shown with blue glyphs. Arrows indicate the strand of the gene annotation, and different levels of shading correspond to different gene structure features: dark shading for coding sequence, medium shading for UTRs, and light shading for introns. Green brackets denote gene loci as determined by the common practice of using only the genomic coordinates from reference gene annotations.Orange brackets denote gene loci as determined by ParsEval, which takes into account both reference and prediction annotations when selecting distinct loci for comparison

One common approach involves designating one set of annotations as the *reference* set and then using the coordinates of each reference gene annotation to define a distinct gene locus to serve as the basis for subsequent comparison (see Figure 1). However, this approach is unfavorable for several related reasons. First, reference gene annotations that overlap are handled separately, when it makes more sense to associate them with the same locus and handle them together. Second, it forces a quality judgment between the two sets of annotations when their relative quality is often unknown. The two sets of annotations likely include complementary information, and unless there is a clear distinction in quality between the two, choosing one as a reference discards clearly related information from the other. Third, relevant information from predicted gene models that extend beyond the boundaries of the corresponding reference annotation is ignored.

**Figure 2:**
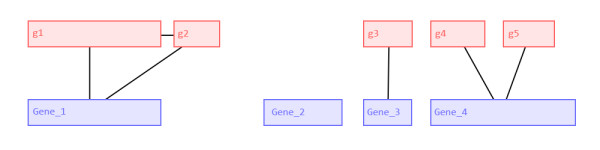
**Locus Identification Using a Gene Interval Graph.** Red and blue nodes in this interval graph correspond to reference and prediction gene annotations (respectively) as shown in Figure 1. Two nodes are connected by an edge if the corresponding gene annotations overlap. Each connected component in the graph represents a distinct *gene locus*, defined as the smallest genomic region containing every gene annotation associated with the corresponding subgraph. In this example, nodes representing five reference annotations and four prediction annotations are shown. The four connected components in the graph correspond to four gene loci, for which precise genomic coordinates can be determined from the associated genes (shown in orange brackets in Figure 1)

Although ParsEval uses the terms *reference* and *prediction* to distinguish between the two sets of annotations, both are considered equally when identifying gene loci. Each gene annotation corresponds to a node in an interval graph *G*. There is an edge between two nodes _*Gi*_and _*Gj*_ if the corresponding gene annotations overlap (see Figure 2). Each connected component in *G* then corresponds to a distinct gene locus, which we define as the smallest genomic region containing every gene annotation associated with the corresponding subgraph. Defining a gene locus in this way makes no assumptions as to the relative quality of the two sets of annotations, and ensures that no potentially relevant data are discarded. Furthermore, according to this definition each gene locus is independent, enabling the subsequent comparative analysis tasks to run in parallel.

### Gene structure representation

To facilitate analysis at each gene locus, ParsEval converts GFF3 annotations for each gene into a character string representing the annotated gene structure (a *model vector*). This model vector is similar to a sequence in Fasta format, except instead of using the alphabet {*A, C, G, T*} to represent chemical composition at each nucleotide, the alphabet {*C, F, G, I, T*} representing gene structure is used: *C* for coding sequence, *F* for 5’-UTR, *T* for 3’-UTR, *I* for introns, and *G* for intergenic sequence. Using this alphabet, each transcript can be represented by a single model vector. ParsEval uses these model vectors when comparing reference and prediction gene annotations.

In many cases, a single pair of model vectors (one for the reference, one for the prediction) is sufficient to fully represent annotated gene structure at a given locus. This is certainly true when both the reference and the prediction annotate a single gene with a single mRNA product at the locus. But even if the reference (or the prediction) annotates multiple genes or transcripts, non-overlapping annotations can be encoded in the same model vector and compared simultaneously with corresponding annotations from the other data set. However, if either the reference or the prediction contains annotations for overlapping transcripts, either because of alternative splicing or because of overlapping gene models, a single pair of model vectors is insufficient to represent the complete annotated gene structure at that locus. In these more complicated cases, the reference or the prediction or both will be associated with multiple model vectors. Thus, the algorithmic requirement is to represent all annotated transcript structures in the locus using the smallest number of model vectors.

This problem reduces to a common problem in graph theory known as the *maximal clique enumeration problem*[9]. We treat each transcript as a node in an undirected graph and place an edge between two nodes if the corresponding transcripts do not overlap (unlike the locus identification step, reference annotations and prediction annotations are handled separately in this step). Each maximal clique (maximal fully-connected subgraph) in this graph corresponds to a set of transcripts that do not overlap and can therefore be collapsed into a single model vector. ParsEval uses the Bron-Kerbosch algorithm [9] to enumerate all maximal transcript cliques, first for the reference and then for the prediction. A model vector is generated for each clique, after which ParsEval compares all reference model vectors with all prediction model vectors.

### Comparative analysis of annotations

Given a pair of equal-length model vectors representing a pair of gene structure annotations at a given locus, ParsEval computes a variety of comparison statistics to measure the level of agreement between the pair of annotations. Calculated at different levels of resolution, these statistics provide a detailed assessment of similarity between the reference and the prediction. At the resolution of distinct annotation features, ParsEval calculates the sensitivity and specificity as described in [3], the F1 score as described in [4], and the annotation edit distance as described in [5,10]. These statistics are calculated for exons, CDS segments, and UTR segments. Note that for a prediction feature to be considered a true positive, ParsEval requires both the start and end coordinates to match the reference perfectly.

At the nucleotide-level resolution, ParsEval also calculates the sensitivity, specificity, F1 score, and annotation edit distance, as well as the simple matching coefficient and the correlation coefficient as described in [3]. These statistics are calculated for coding nucleotides (CDS) and untranslated exonic nucleotides (UTR). Overall identity at the nucleotide level, of which the simple matching coefficient is a generalization, is also computed.

For complex loci requiring multiple comparisons, the locus report includes an aggregate summary of the similarity statistics at the locus level in addition to the reports for each individual comparison. This locus-level summary also includes the splice complexity statistic [5], which ParsEval computes and reports for both the reference and the prediction at the locus level.

Based on the computed statistics, each comparison is classified in terms of similarity. A comparison is classified as a *perfect match* if the model vectors (and by implication the annotated gene structures) are identical. A comparison is classified as a *CDS structure match* if the comparison is not a perfect match, but there is perfect agreement in terms of CDS structure. A comparison is classified as an *exon structure match* if there are differences in the coding sequence that nevertheless preserve exon structure (as resulting from different start and/or stop codons). A comparison is classified as a *UTR structure match* if there are differences in CDS and exon structure, but the UTR structures are identical. All other comparisons are classified as *non-matches*.

Note that, as with feature-level statistics, match classifications require perfect agreement. For instance, a pair of annotations may have very similar CDS structures, and this will be reflected in the nucleotide-level CDS statistics. However, if the CDS structures are not precisely identical, the comparison will not be classified as a *CDS structure match*.

As comparison statistics are computed on a locus-by-locus basis, ParsEval also maintains a running total of all comparison counts (such as true positives and false positives) from which the statistics are computed. When all loci have been considered, each comparison statistic is then recomputed using these running totals to provide an overall assessment of similarity.

### Reporting comparison scores

For each gene locus, comparison statistics are calculated for each corresponding pair of reference and prediction model vectors. If multiple comparisons are required at a locus, however, statistics are not reported for each comparison. The comparisons are ranked using the previously described similarity statistics and are reported so as to ensure each transcript (or transcript clique) is considered at most one time. In cases where there is an unequal number of reference and prediction transcripts (or transcript cliques) associated with a particular locus, some will be labeled as novel or unmatched transcripts, and corresponding statistics are not included in ParsEval’s reports.

ParsEval presents the comparison statistics in a collection of reports. The first is a single summary report providing the aggregated statistics for a high-level assessment of similarity, as is standard for tools of this kind. Additionally, ParsEval produces a dedicated comparison report for each individual locus. The detail provided by these locus-level reports is extremely valuable, and ParsEval is the only tool of its kind that preserves and reports comparisons at this level. By default, ParsEval generates these reports in an easy-to-parse and easy-to-read text format. However, ParsEval can also generate the reports as hyperlinked HTML files to facilitate browsing and network-based distribution. Furthermore, ParsEval can supplement HTML reports with embedded PNG graphics providing a genome-browser-like view of each locus’ genomic context and enabling visual assessment of the annotations.

If more targeted reporting is desired, ParsEval also provides some filtering features. Using a simple optional configuration file, the user can exclude some gene loci from the reports based on a variety of features: locus length, number of genes, number of transcripts, number of transcripts per gene, number of exons, and CDS length. No comparisons are performed for loci that are filtered out, and thus do not contribute to the reported aggregate summary statistics and comparison classifications.

**Figure 3:**
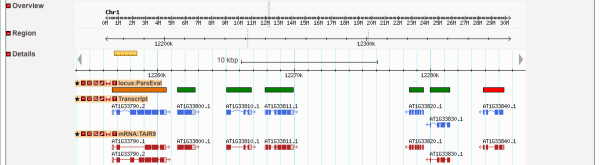
**Integrating ParsEval Reports with a Genome Browser.** Screenshot of the *Arabidopsis thaliana* genome browser at Phytozome (http://phytozome.net/), with a custom anonymous user track populated by ParsEval output. Boxes in this custom track represent loci identified by ParsEval and are color-coded according to the level of agreement between the two sets of annotations compared (dark red and pastel blue glyphs, respectively). This custom track can easily be configured so that features are hyperlinked to ParsEval reports containing detailed comparison statistics

To facilitate integration of comparison reports with popular genome browsers such as GBrowse [11] and PlantGDB [12], ParsEval can generate an additional output file (in GFF3 format) containing the coordinates of each gene locus. These genome browsers commonly allow users to anonymously create private custom tracks with uploaded data, which provides the quickest mechanism for integration. Once a track is populated with the uploaded locus data, the user can edit the track configuration so that each locus feature in the track is hyperlinked to the corresponding ParsEval report, which may have been stored, for example, on that user’s local machine (see Figure 3). Alternatively, if a more permanent and public solution is desired, a user with administrative privileges for the genome browser can follow standard procedures for populating a new track with the GFF3 data and then configure the track so that locus features are linked to network-accessible ParsEval reports.

## Results and discussion

We present several use cases to demonstrate ParsEval’s capabilities, benchmark its performance, and compare its utility relative to existing methods. The input data for these demonstrations were obtained from a variety of public databases with different respective formatting conventions. Accordingly, all data files were processed and converted to a uniform format before analysis. A detailed description of this conversion process, along with all code and commands used, are provided in the Additional file 1 as well as in ParsEval’s source code distribution.

Unless otherwise noted, all use cases and benchmarks described herein were run on a fairly modest desktop computer: a Mac Pro with two 2.8 GHx quad-core Intel Xeon processors and 4 GB of RAM. ParsEval’s performance for these demonstrations should therefore be fairly representative of the performance one might expect when running on commodity laboratory or personal hardware.

### Use case: predictions vs. gold standard

High-quality gene structure annotations derived from a combination of computational and experimental evidence, and possibly improved with expert manual curation, are indispensably used as “gold standards” for measuring the accuracy of a novel gene prediction method or entire new annotation workflows. Identifying differences between the new method’s predictions and such gold standard reference can help identify areas in which the novel method provides or needs improvement. Reports from ParsEval are effective for quickly and clearly identifying such differences.

To demonstrate ParsEval in this context, we reproduced a comparison that was originally published to assess the performance of the AUGUSTUS gene prediction program [13]. In the original study, AUGUSTUS was tested on the *h178* data set [14], a set of 178 human genomic sequences, each containing a single gene, for which annotations were available from the EMBL database release 50 [15]. Gene predictions from AUGUSTUS were compared the annotations from EMBL, and sensitivity and specificity scores were calculated at the nucleotide level, the exon level, and the gene level.

We obtained the *h178* data set (sequences and EMBL r50 annotations) from [16]. We then used the latest version of AUGUSTUS (2.5.5) to generate gene predictions for the 178 sequences. The data files were reformatted and then compared using ParsEval. Running on a desktop computer, ParsEval generated graphical reports in less than a minute. The summary report provided immediate access to a variety of similarity metrics, including those reported in the original assessment. The sensitivity and specificity values reported by ParsEval are comparable to those reported in the original AUGUSTUS manuscript (see Table 2). Differences in the comparison metrics can likely be explained by improvements to the AUGUSTUS program since publication, although the exact reason is elusive because the original AUGUSTUS software is no longer accessible.

**Table 2:** Annotation comparison methods

**Statistic**	**AUGUSTUS**	**ParsEval**			
	**manuscript**	**comparison**			
Coding nucleotide sensitivity	0.93	0.94			
Coding nucleotide specificity	0.90	0.99			
Exon sensitivity	0.80	0.81			
Exon specificity	0.81	0.86			
Gene sensitivity	0.48	0.43			
Gene specificity	0.47	0.46			

Sensitivity and specificity scores for AUGUSTUS gene predictions in comparison to corresponding gene annotations from EMBL database release 50. The first column shows scores as reported in the original AUGUSTUS manuscript. The second column shows scores as computed by ParsEval using predictions from the latest version of AUGUSTUS (2.5.5). Summary reports from ParsEval provide immediate access to a wide variety of similarity statistics, including the ones reported in this table. Differences between the scores reported by the AUGUSTUS authors and the ParsEval authors are likely due to subsequent updates of the AUGUSTUS program since its publication.

### Use case: two sets of annotations

When working with genome annotations, there is an increasing variety of cases in which no gold standard is available for comparison. For example, gene annotations for many model species are available from a variety of sources (i.e., UCSC versus Ensembl). The respective quality of these different annotation sets is not always clear, but comparison is still a necessary and fundamental task. Another example relates to genome projects that typically offer multiple releases of gene annotations between each major genome assembly release. Although newer releases may offer marginal improvements over the older ones, neither one can truly be considered a high-quality standard reference for comparison. An additional example relates to the increased affordability of genome sequencing and the number of new and exotic species for which genome sequence is available. Gene annotation software is based on complex statistical models containing many parameters, and it is not always initially clear which parameter values to use up front. Therefore, when annotating a newly sequenced genome, it is common to extract a subset of the genome on which to perform repeated optimization runs to determine the parameter values that should be used subsequently to annotate the entire genome.

In each of these scenarios, multiple annotation sets must be compared, despite having no intuition as to the relative quality of the respective annotations. ParsEval was designed precisely for this type of analysis. Reports from ParsEval provide both an overall summary and locus-level detail, enabling the user to make informed decisions about annotations for individual loci, as well as for annotation sets as a whole.

As a demonstration of ParsEval’s capability in this context, we downloaded two recent gene annotation releases (releases 64 and 65) for *Mus musculus* from the Ensembl database [17]. We compared these annotations using ParsEval, which required approximately 3 minutes of runtime on a desktop computer. A brief review of ParsEval’s summary report shows that a total of 20,362 gene loci were identified using these annotations (see Table 3 for a complete breakdown). Of these gene loci, 6,725 had only annotations from release 64.

**Table 3:** Annotation comparison methods

Perfect matches	22,333	94.7%			
CDS structure matches	0	0.0%			
Exon structure matches	0	0.0%			
UTR structure matches	83	0.4%			
Non-matches	1,174	5.0%			
**Total comparisons**	23,590	100.0%			

Results from a ParsEval comparison of gene annotations for *Mus musculus* from two recent releases of the Ensembl database (releases 64 and 65). Release 64 contains 22,507 gene annotations, while release 65 contains 14,486 gene annotations. ParsEval identified 20,362 gene loci using these two data sets, 6,725 of which contained only annotations from release 64. For the 13,637 gene loci for which both release 64 and 65 have annotations, 23,590 comparisons were performed. Each of these comparisons was classified according to how well the annotations from the two releases agreed. This table shows a breakdown of these results.

23,590 comparisons were performed by ParsEval, of which 22,333 (94.7%) were perfect matches between releases 64 and 65. A small number (83, 0.4%) of comparisons were classified as UTR structure matches. For the remaining 1,174 comparisons (5.0%) that were classified as non-matches, transcripts from release 64 contained an average of 16.47 exons, whereas transcripts from release 65 contained an average of 8.11 exons. A brief review of a handful of selected loci showed that many long transcripts (with many exons) that had been present in release 64 were absent in release 65.

This use case is an ideal demonstration of ParsEval’s capabilities. Although the authors have no prior experience working with these particular data sets, a cursory examination ParsEval’s reports clearly draw attention to an important fact—between release 64 and 65, changes to Ensembl’s annotation pipeline (perhaps different values for parameters that influence joining/splitting annotations, or implementation of stricter filters for gene length) affected approximately 5% of the gene annotations. Not only does ParsEval provide this information in a summarized form, it also provides detailed locus reports enabling users to scrutinize the results on a gene-by-gene basis. This breadth and detail of information is of great benefit to a wide variety of scientists and will empower them to more fully understand the available data and make informed decisions regarding alternative sources of annotation.

### Benchmarks

To demonstrate its speed, scalability, and efficiency, we benchmarked ParsEval by analyzing pairs of whole-genome gene structure annotations for four common model organisms representing a wide range of eukaryotic diversity: *Arabidopsis thaliana* (thale cress), *Drosophila melanogaster* (fruit fly), *Glycine max* (soybean), and *Homo sapiens* (human) (see Table 4). To give a detailed demonstration of its performance, ParsEval was run 24 times for each species—3 technical replicates while varying the output mode (text and HTML/PNG) and the number of dedicated processors (1, 2, 4, and 8). Reported runtimes were obtained by taking the mean of the 3 corresponding replicates.

**Table 4:** Annotation comparison methods

	***A. thaliana***		***D. melanogaster***		***G. max***		***H. sapiens***	
**Reference annotations**	TAIR9		FlyBase 5.39		NCBI Entrez		UCSC knownGene (hg19)	
**Prediction annotations**	TAIR10		Ensembl r65		JGI / Phytozome		Ensembl r65	
**Average runtime (sec)**	**Text**	**HTML**	**Text**	**HTML**	**Text**	**HTML**	**Text**	**HTML**
*n*=1	36.3	859.4	91.1	1,350.5	85.3	1,461.1	294.3	6,422.0
*n*=2	32.8	449.2	56.6	859.5	79.4	768.4	181.3	4,089.5
*n*=4	30.7	246.5	39.2	633.7	76.5	439.9	130.1	2,751.2
*n*=8	29.8	168.7	32.4	546.6	76.3	330.5	108.0	2,323.3
**Gene loci**	25,618		10,976		47,877		17,865	
**shared**	25,590		10,944		37,942		7,779	
**unique to reference**	6		32		3,363		9,569	
**unique to prediction**	22		0		6,572		517	
**Comparisons**	33,002		22,474		38,734		16,168	
**perfect matches**	31,750	96.2%	22,446	99.9%	2,489	6.4%	2,517	15.6%
**CDS structure matches**	420	1.3%	0	0.0%	17,450	45.1%	8,269	51.1%
**exon structure matches**	8	0.0%	21	0.1%	26	0.1%	27	0.2%
**UTR structure matches**	159	0.5%	1	0.0%	647	1.7%	58	0.4%
**non-matches**	665	2.0%	6	0.0%	18,122	46.8%	5,297	32.8%

#### Performance in text output mode

ParsEval demonstrated optimal performance when running in text output mode, with runtimes ranging between about 30 seconds to about 4 minutes. Running ParsEval in parallel on multiple processors provided noticeable improvement in runtime for *Drosophila* and human, although no improvement was seen for *Arabidopsis* and soybean. It is likely that for loci with relatively small and simple gene structures, ParsEval’s runtime is bound more by serial I/O related tasks than by actual analytical computations, which would explain why no improvement was observed for the plant species.

#### Performance in HTML output mode with PNG graphics

Running ParsEval in HTML/PNG output mode increased the runtimes by an order of magnitude, although parallel processing kept these runtimes within a reasonable range (about a half hour for the most intensive comparison) with observed speedup factors ranging from 3 to 5 when using all 8 processors. Because these improvements in runtime were observed for all species, it is likely that ParsEval’s runtime is bound primarily by computationally intensive graphics generation tasks when running in HTML/PNG output mode.

#### Notes on benchmark results

The results of the *A. thaliana* benchmark were not surprising. Perfect matches and CDS matches account for 97.5% of the comparisons, which makes sense considering that TAIR10 represents minor cumulative updates to TAIR9. There were even fewer differences between FlyBase and Ensembl annotations for the *D. melanogaster* benchmark (≈ 0.1% of loci), suggesting perhaps that these differences may be the consequence of technical artifacts in one data set or the other.

The results of the other two benchmarks, for *G. max* and *H. sapiens*, were somewhat surprising. In each case, approximately 10% of the comparisons reflected perfect matches between the two annotations (6.4% for soybean and 15.3% for human), while approximately 50% of the comparisons reflected CDS matches (45.1% for soybean and 54.9% for human). Therefore, for the remaining approximate 30% of human genes and 50% of soybean genes, the annotated coding sequences (and associated polypeptides) are different depending on the annotation source. These differences are likely the result of different annotation strategies between alternative annotation approaches. Until the problem of gene structure prediction is completely solved, alternative approaches yielding alternative results will be inevitable. The ParsEval tool will aid both producers and users of gene structure annotations to quickly assess the extent and nature of the approach-based differences.

### Performance evaluation in comparison to Eval software

To evaluate ParsEval’s performance in comparison to existing methods, we used the Eval tool [1] to repeat one of the previously described use cases. Gene annotations for *Mus musculus* were retrieved from releases 64 and 65 of the Ensembl database, and subsequently analyzed using both Eval and ParsEval. Some small differences were observed in the similarity statistics computed by the two programs, although this was not unexpected as Eval uses a different approach than ParsEval for matching reference annotations to prediction annotations. Also, the two programs provide a different breakdown of the similarity statistics, making a rigorous comparison between the Eval results and the ParsEval results impractical.

Running Eval on the complete data sets exhausted the desktop computer’s memory resources after several minutes, so comparison of Eval and ParsEval was only possible after restricting the data sets to annotations for *M. musculus* chromosomes 1 through 10. To analyze these reduced data sets, Eval required an average of 12 minutes 13 seconds and consumed all available memory. On the other hand, ParsEval, running on a single processor, required an average of 1 minute 44 seconds, with memory consumption peaking at approximately 0.5 GB. When run on 4 processors, ParsEval’s performance margin increased with an average runtime of 47 seconds.

To ensure that Eval’s performance was not being severely affected by the desktop’s limited system memory, the comparison was also performed in a high-performance computing environment in which memory could not have been a limiting factor. ParsEval continued to demonstrate superior performance in this environment as well, although by a slightly less drastic margin. The Eval program required an average of 7 minutes 18 seconds of runtime, while ParsEval required an average of 1 minute 19 seconds using a single processor, or 37 seconds using 4 processors.

These tests conclusively demonstrate two important points regarding the performance of ParsEval relative to Eval: not only is ParsEval markedly faster, but its resource efficiency also makes it much better equipped to run whole-genome comparisons on the laptop or desktop computers one might expect to see in the typical biology lab. The initial runtimes reported herein should be fairly representative of what users can expect to observe when running ParsEval on commodity hardware.

## Conclusions

The accessibility of genome annotation tools to an increasingly wider variety of scientists will soon be accompanied by an increased demand for supplementary tools to manage and analyze genome annotations. We address this need with ParsEval, a tool for fulfilling a common, fundamental analytical need for which existing software is lacking. ParsEval is a portable, easy-to-install, and efficient program for comparing gene structure annotations, and facilitates a wide variety of downstream comparative analyses. We demonstrate the speed and scalability of ParsEval, even when working with large eukaryotic genomes. Furthermore, we highlight the capability of the detailed comparison statistics in ParsEval reports to highlight relevant biological trends in the data. We anticipate that ParsEval will enable a wide variety of biologists to more fully take advantage of the vast genome annotation data resources accumulating in their individual labs and in the community at large.

## Availability and requirements

· Project name: ParsEval

· Project home page:http://parseval.sourceforge.net

· Operating system(s): POSIX-compliant UNIX systems (Linux, Mac OS X, Cygwin, Solaris, etc.)

· Programming language: ANSI C

· Other requirements: C compiler with OpenMP support (such as GCC 4.2 or higher), GenomeTools library http://genometools.org

· License: ISC

· Any restrictions to use by non-academics: none

## Competing interests

The authors declare that they have no competing interests.

## Author’s contributions

DS designed and implemented the software and drafted the manuscript. VB supervised the project and provided design and feature suggestions. Both authors conceived the project, edited the manuscript, and approved the final version.

## Funding

This work was supported in part by the U.S.A. National Science Foundation Plant Genome Research Program grant ISO#1126267 to V.B..

## Supplementary Material

Additional file 1 **Supplemental data.** The file StandageBrendel-7-6-12-SupplementalData.tar.gz is a gzip-commpressed tar archive that stores a self-contained web page. This page includes supplemental information for users regarding the use cases and benchmarks described in the paper, providing detailed instructions for obtaining the corresponding data and code for carrying out the use cases and benchmarks. Click here for file
