# Supplementary material for: ParsEval: parallel comparison and analysis of gene structure annotations
Source: BMC Bioinformatics. 2012 Aug 1;13:187. doi: 10.1186/1471-2105-13-187 (PMC3439248; doi:10.1186/1471-2105-13-187)
Supplement: Additional file 1 — Supplemental data. The file StandageBrendel-7-6-12-SupplementalData.tar.gz is a gzip-commpressed tar archive that stores a self-contained web page. This page includes supplemental information for users regarding the use cases and benchmarks described in the paper, providing detailed instructions for obtaining the corresponding data and code for carrying out the use cases and benchmarks. [file 1471-2105-13-187-S1.gz › supplemental-data/index.html]

research:parseval:supplement


−Table of Contents

- Supplementary data

  - Running use cases
  - Running benchmarks
  - Building data files

# Supplementary data

The input data for ParsEval's use cases and benchmarks were obtained from a variety of public databases with different respective formatting conventions.
Accordingly, all data files were processed and converted to a uniform format before analysis.
Here we provide a detailed description of this conversion process, along with all code and commands used to run the analyses and conversions.

## Running use cases

The following script is an example of how the use cases were run.
It can be run using the command `bash run-usecases.sh 1`, where you can replace **1** with the number of available processors.
Corresponding data files can be found in ParsEval's supplementary data, or in the code distribution itself.

run-usecases.sh
:   ```
    #!/bin/bash
    parseval -d -e both -f html -g -i both -n $1 -o h178 -p -v h178-refr-embl50.gff3.bz2 h178-pred-augustus.gff3.bz2 2> h178.log
    parseval -d -g -i both -n $1 -o mmus.txt -u both -v mmus-refr-ensembl64.gff3.bz2 mmus-pred-ensembl65.gff3.bz2 2> mmus.log
    parseval -d -g -i both -o mmus-parseval.txt -u both -v mmus-refr-ensembl64-subset.gff3.bz2 mmus-pred-ensembl65-subset.gff3.bz2 2> mmus-parseval.log
    evaluate_gtf.pl -v mmus-refr-ensembl64-subset.gtf mmus-pred-ensembl65-subset.gtf > mmus-eval.txt 2> mmus-eval.log
    ```

## Running benchmarks

The following script is a simplified example of how the benchmarks were run.
It can be run using the command `run-benchmarks.sh 1`, where you can replace **1** with the number of available processors.
Corresponding data files can be found in ParsEval's supplementary data, or in the code distribution itself.

run-benchmarks.sh
:   ```
    #!/bin/bash
    parseval -d -g -i both -n $1 -o athal.txt -v athal-refr-tair9.gff3.bz2 athal-pred-tair10.gff3.bz2 2> athal.log
    parseval -d -g -i pred -n $1 -o dmel.txt -u pred -v dmel-refr-flybase.gff3.bz2 dmel-pred-ensembl.gff3.bz2 2> dmel.log
    parseval -d -e pred -g -i both -n $1 -o gmax.txt -u refr -v gmax-refr-entrez.gff3.bz2 gmax-pred-jgi.gff3.bz2 2> gmax.log
    parseval -d -g -i both -n $1 -o hsap.txt -u both -v hsap-refr-ucsc.gff3.bz2 hsap-pred-ensembl.gff3.bz2 2> hsap.log
    ```

The *actual* code used by the authors to run the benchmarks (complete with technical replicates, etc) is shown below.

```
# Benchmarks in 'text' output mode
for x in 1 2 4 8
do
  for y in a b c
  do
    parseval -d -g -i both -n $x -o athal-$x$y.txt -v athal-refr-tair9.gff3.bz2 athal-pred-tair10.gff3.bz2 2> athal-$x$y-txt.log
  done
done
 
for x in 1 2 4 8
do
  for y in a b c
  do
    parseval -d -g -i pred -n $x -o dmel-$x$y.txt -u pred -v dmel-refr-flybase.gff3.bz2 dmel-pred-ensembl.gff3.bz2 2> dmel-$x$y-txt.log
  done
done
 
for x in 1 2 4 8
do
  for y in a b c
  do
    parseval -d -e pred -g -i both -n $x -o gmax-$x$y.txt -u refr -v gmax-refr-entrez.gff3.bz2 gmax-pred-jgi.gff3.bz2 2> gmax-$x$y-txt.log
  done
done
 
for x in 1 2 4 8
do
  for y in a b c
  do
    parseval -d -g -i both -n $x -o hsap-$x$y.txt -u both -v hsap-refr-ucsc.gff3.bz2 hsap-pred-ensembl.gff3.bz2 2> hsap-$x$y-txt.log
  done
done
 
 
# Benchmarks in 'html' output mode
for x in 1 2 4 8
do
  for y in a b c
  do
    parseval -d -f html -p -g -i both -n $x -o athal-$x$y-html -v athal-refr-tair9.gff3.bz2 athal-pred-tair10.gff3.bz2 2> athal-$x$y-html.log
  done
done
 
for x in 1 2 4 8
do
  for y in a b c
  do
    parseval -d -f html -p -g -i pred -n $x -o dmel-$x$y-html -u pred -v dmel-refr-flybase.gff3.bz2 dmel-pred-ensembl.gff3.bz2 2> dmel-$x$y-html.log
  done
done
 
for x in 1 2 4 8
do
  for y in a b c
  do
    parseval -d -f html -p -e pred -g -i both -n $x -o gmax-$x$y-html -u refr -v gmax-refr-entrez.gff3.bz2 gmax-pred-jgi.gff3.bz2 2> gmax-$x$y-html.log
  done
done
 
for x in 1 2 4 8
do
  for y in a b c
  do
    parseval -d -f html -p -g -i both -n $x -o hsap-$x$y-html -u both -v hsap-refr-ucsc.gff3.bz2 hsap-pred-ensembl.gff3.bz2 2> hsap-$x$y-html.log
  done
done
```

## Building data files

The following makefile was used to build the data files.
With the exception of the Entrez annotations for *Glycine max*,
this build file will download all data directly from the primary source.
The Entrez data are not easily accessible through a command-line
interface, so they are provided in ParsEval's source code distribution,
along with all of the scripts referenced in the makefile.

makefile
:   ```
    PE_COMPILE_DIR=/usr/local/src/parseval
    PERL_SOURCE=$(PE_COMPILE_DIR)/data/benchmarks/build
    GMAX_XML_SOURCE=$(PERL_SOURCE)
     
    all:	athal dmel gmax h178 hsap mmus
     
    athal:	athal-pred-tair10.gff3.bz2 athal-refr-tair9.gff3.bz2
     
    dmel:	dmel-pred-ensembl.gff3.bz2 dmel-refr-flybase.gff3.bz2
     
    gmax:	gmax-pred-jgi.gff3.bz2 gmax-refr-entrez.gff3.bz2
     
    h178:	h178-pred-augustus.gff3.bz2 h178-refr-embl50.gff3.bz2
     
    hsap:	hsap-pred-ensembl.gff3.bz2 hsap-refr-ucsc.gff3.bz2
     
    mmus:	mmus-pred-ensembl65.gff3.bz2 mmus-refr-ensembl64.gff3.bz2
     
    athal-pred-tair10.gff3.bz2:	
    				wget ftp://ftp.arabidopsis.org/home/tair/Genes/TAIR10_genome_release/TAIR10_gff3/TAIR10_GFF3_genes.gff
    				gt gff3 -force -retainids -sort -tidy -bzip2 -o athal-pred-tair10.gff3.bz2 TAIR10_GFF3_genes.gff
    				rm TAIR10_GFF3_genes.gff
     
    athal-refr-tair9.gff3.bz2:	
    				wget ftp://ftp.arabidopsis.org/home/tair/Genes/TAIR9_genome_release/TAIR9_gff3/TAIR9_GFF3_genes.gff
    				gt gff3 -force -retainids -sort -tidy -bzip2 -o athal-refr-tair9.gff3.bz2 TAIR9_GFF3_genes.gff
    				rm TAIR9_GFF3_genes.gff
     
    dmel-pred-ensembl.gff3.bz2:	
    				wget ftp://ftp.ensembl.org/pub/release-65/gtf/drosophila_melanogaster/Drosophila_melanogaster.BDGP5.25.65.gtf.gz
    				zcat Drosophila_melanogaster.BDGP5.25.65.gtf.gz | perl $(PERL_SOURCE)/ensembl_gtf_to_gff3.pl stopcodon2cds | perl $(PERL_SOURCE)/mergecds.pl > dmel-ensembl.gff3
    				gt gff3 -force -retainids -sort -tidy -bzip2 -o dmel-pred-ensembl.gff3.bz2 dmel-ensembl.gff3
    				rm dmel-ensembl.gff3 Drosophila_melanogaster.BDGP5.25.65.gtf.gz
     
    dmel-refr-flybase.gff3.bz2:	
    				wget ftp://ftp.flybase.net/releases/FB2011_07/dmel_r5.39/gff/dmel-all-r5.39.gff.gz
    				zcat dmel-all-r5.39.gff.gz | perl $(PERL_SOURCE)/flybase-sort.pl > dmel-sorted.gff3
    				gt gff3 -force -retainids -sort -tidy -bzip2 -o dmel-refr-flybase.gff3.bz2 dmel-sorted.gff3
    				rm dmel-all-r5.39.gff.gz dmel-sorted.gff3
     
    gmax-pred-jgi.gff3.bz2:		
    				wget ftp://ftp.jgi-psf.org/pub/JGI_data/phytozome/v7.0/Gmax/annotation/Gmax_109_gene.gff3.gz
    				gt gff3 -force -retainids -sort -tidy -bzip2 -o gmax-pred-jgi.gff3.bz2 Gmax_109_gene.gff3.gz
    				rm Gmax_109_gene.gff3.gz
     
    gmax-refr-entrez.gff3.bz2:	
    				bzcat $(GMAX_XML_SOURCE)/gmax-entrez.xml.bz2 | php $(PERL_SOURCE)/ncbixml-to-gff3.php | php $(PERL_SOURCE)/gmax-chr-rename.php > gmax-renamed.gff3
    				gt gff3 -force -retainids -sort -tidy -bzip2 -o gmax-refr-entrez.gff3.bz2 gmax-renamed.gff3
    				rm gmax-renamed.gff3
     
    h178-pred-augustus.gff3.bz2:	
    				wget http://genome.imim.es/datasets/gpeval2000/data/SGS/embl50.h178.fa
    				augustus --species=human --gff3=on embl50.h178.fa > augustus-h178-gff3.txt
    				grep -v '^#$$' augustus-h178-gff3.txt | grep -v '^# ' | grep -v codon | sed 's|transcript|mRNA|' | gt gff3 -force -retainids -sort -tidy -bzip2 -o h178-pred-augustus.gff3.bz2
    				rm embl50.h178.fa augustus-h178-gff3.txt
     
    h178-refr-embl50.gff3.bz2:	
    				wget http://genome.imim.es/datasets/gpeval2000/data/SGS/embl50.h178.gff
    				perl $(PERL_SOURCE)/h178-gff_to_gff3.pl < embl50.h178.gff > embl-h178.gff3
    				gt gff3 -force -retainids -sort -tidy -bzip2 -o h178-refr-embl50.gff3.bz2 embl-h178.gff3
    				rm embl50.h178.gff embl-h178.gff3
     
    hsap-pred-ensembl.gff3.bz2:	
    				wget ftp://ftp.ensembl.org/pub/release-63/gtf/homo_sapiens/Homo_sapiens.GRCh37.63.gtf.gz
    				zcat Homo_sapiens.GRCh37.63.gtf.gz | perl $(PERL_SOURCE)/ensembl_gtf_to_gff3.pl > hsap-ensembl.gff3
    				gt gff3 -force -retainids -sort -tidy -bzip2 -o hsap-pred-ensembl.gff3.bz2 hsap-ensembl.gff3
    				rm Homo_sapiens.GRCh37.63.gtf.gz hsap-ensembl.gff3
     
    hsap-refr-ucsc.gff3.bz2:	
    				genePredToGtf hg19 knownGene knownGene.gtf
    				perl $(PERL_SOURCE)/ucsc_gtf_to_gff3.pl < knownGene.gtf > knownGene.gff3
    				gt gff3 -force -retainids -sort -tidy -bzip2 -o hsap-refr-ucsc.gff3.bz2 knownGene.gff3
    				rm knownGene.gtf knownGene.gff3
     
    mmus-pred-ensembl65.gff3.bz2:	
    				wget ftp://ftp.ensembl.org/pub/release-65/gtf/mus_musculus/Mus_musculus.NCBIM37.65.gtf.gz
    				zcat Mus_musculus.NCBIM37.65.gtf.gz | perl -ne '$$line = $$_; if($$line =~ m/(.+)\tprotein_coding\t/){ $$seq = $$1; if(not $$seq =~ m/^\D/ and $$seq < 11){ printf("chr%s", $$line);}}' > mmus-pred-ensembl65-subset.gtf
    				zcat Mus_musculus.NCBIM37.65.gtf.gz | perl $(PERL_SOURCE)/ensembl_gtf_to_gff3.pl > mmus-65.gff3
    				gt gff3 -force -retainids -sort -tidy -bzip2 -o mmus-pred-ensembl65.gff3.bz2 mmus-65.gff3
    				bzcat mmus-pred-ensembl65.gff3.bz2 | perl -ne 'print if(m/^chr(\d+)\t/ and $$1 < 11);' | gt gff3 -retainids -sort -tidy -bzip2 -o mmus-pred-ensembl65-subset.gff3.bz2
    				rm Mus_musculus.NCBIM37.65.gtf.gz mmus-65.gff3
     
    mmus-refr-ensembl64.gff3.bz2:	
    				wget ftp://ftp.ensembl.org/pub/release-64/gtf/mus_musculus/Mus_musculus.NCBIM37.64.gtf.gz
    				zcat Mus_musculus.NCBIM37.64.gtf.gz | perl -ne '$$line = $$_; if($$line =~ m/(.+)\tprotein_coding\t/){ $$seq = $$1; if(not $$seq =~ m/^\D/ and $$seq < 11){ printf("chr%s", $$line);}}' > mmus-refr-ensembl64-subset.gtf
    				zcat Mus_musculus.NCBIM37.64.gtf.gz | perl $(PERL_SOURCE)/ensembl_gtf_to_gff3.pl > mmus-64.gff3
    				gt gff3 -force -retainids -sort -tidy -bzip2 -o mmus-refr-ensembl64.gff3.bz2 mmus-64.gff3
    				bzcat mmus-refr-ensembl64.gff3.bz2 | perl -ne 'print if(m/^chr(\d+)\t/ and $$1 < 11);' | gt gff3 -retainids -sort -tidy -bzip2 -o mmus-refr-ensembl64-subset.gff3.bz2
    				rm Mus_musculus.NCBIM37.64.gtf.gz mmus-64.gff3
    ```
